# Reconstruction of an SSR-based *Magnaporthe oryzae* physical map to locate avirulence gene *AvrPi12*

**DOI:** 10.1186/s12866-018-1192-x

**Published:** 2018-05-31

**Authors:** Tonghui Li, Jianqiang Wen, Yaling Zhang, James Correll, Ling Wang, Qinghua Pan

**Affiliations:** 1 State Key laboratory for Conservation and Utilization of Subtropic Agrobioresurces, Guangdong Provincial Key Laboratory for Crop Molecular Breeding, College of Agriculture, South China Agricultural University, Guangzhou, 510642 China; 2 College of Agronomy, Heilongjiang Bayi Agricultural University, Daqing, 163319 China; 3 Department of Plant Pathology, University of Arkansas, Fayetteville, AR 72701 USA

**Keywords:** *Magnaporthe oryzae*, SSR physical map, Avirulence gene, *AvrPi12*, Genetic and physical mapping

## Abstract

**Background:**

Pathogen avirulence (*Avr*) genes can evolve rapidly when challenged by the widespread deployment of host genes for resistance. They can be effectively isolated by positional cloning provided a robust and well-populated genetic map is available.

**Results:**

An updated, SSR-based physical map of the rice blast pathogen *Magnaporthe oryzae* (*Mo*) has been constructed based on 116 of the 120 SSRs used to assemble the last map, along with 18 newly developed ones. A comparison between the two versions of the map has revealed an altered marker content and order within most of the *Mo* chromosomes. The avirulence gene *AvrPi12* was mapped in a population of 219 progeny derived from a cross between the two *Mo i*solates CHL42 and CHL357. A bulked segregant analysis indicated that the gene was located on chromosome 6, a conclusion borne out by an analysis of the pattern of segregation shown by individual isolates. Six additional PCR-based markers were developed to improve the map resolution in the key region. *AvrPi12* was finally located within the sub-telomeric region of chromosome 6, distal to the SSR locus LSM6–5.

**Conclusions:**

The improved SSR-based linkage map should be useful as a platform for gene mapping and isolation in *Mo*. It was used to establish the location of *AvrPi12*, thereby providing a starting point for its positional cloning.

**Electronic supplementary material:**

The online version of this article (10.1186/s12866-018-1192-x) contains supplementary material, which is available to authorized users.

## Background

The pathogen responsible for the highly damaging disease of rice known as blast is the filamentous ascomycete *Magnaporthe oryzae* (*Mo*) [1, 2]. Deployment of host genes conferring resistance is widely recognized as the most environmentally benign and cost-effective means of its control [3–5]. However, the effectiveness of most blast resistance genes ceases after a few seasons, as a result of the emergence of pathogen races in which the matching avirulence (*Avr*) gene has mutated to virulence [6–10]. A better understanding of the mode of evolution of pathogen *Avr* genes should aid in forming an effective strategy for resistance gene deployment.

Positional cloning has proven to be an effective means of isolating both host resistance and pathogen *Avr* genes [10–14]. The method relies on the prior establishment of a comprehensive linkage map [11, 15]. The effort to develop such a linkage map for *Mo*, begun in the 1990s, and by 2007 had delivered one based on SSR (simple sequence repeats; also called microsatellite) markers, taking advantage of the acquisition of the genome sequence of *Mo* isolate 70–15 (MG5; http://www.ncbi.nlm.nih.gov/bioproject/13840; [15, 16]). In the meantime, improvements have been made to this genome sequence (MG8; www.ncbi.nlm.nih.gov/assembly/GCF_000002495.2/); [17, 18]), which now allows for an update of the *Mo* linkage map.

*Pi12*, a gene which conditions blast resistance, was initially identified in the African cultivar Moroberekan [19, 20], but is also effective in parts of East Asia [21–23]. Here, the updated *Mo* physical map was used to identify the genomic location of the *Avr* gene matching *Pi12,* the most important blast resistance gene harbored by Moroberekan, as a prelude to undertaking its positional cloning.

## Methods

### The *Mo* mapping population

Isolates CHL357 and CHL42 were collected from diseased plants in, respectively, Jiangsu and Yunnan provinces (China). The two isolates were crossed with one another in vitro using a published protocol [24, 25], and the resulting single-spored progeny were incubated for the first 7 days at 25 °C and thereafter at 20 °C under continuous illumination provided by dark-blue fluorescent light. Single ascospores were randomly selected [24], generating a population of 219 viable isolates, which were stored on dry filter papers [25]. This set of progeny represented the mapping population used to determine the genetic location of *AvrPi12.*

### Reconstruction of SSR physical map for *Mo* genome

The SSR loci previously developed based on the genome of the version MG5 [15] were reassembled onto the version MG8 by aligning their primer sequences using the Blast algorithm (http://www.ncbi.nlm.nih.gov/BLAST), even though some primer sequences were slightly improved for better detection. A number of additional SSR assays were subsequently developed to fill gaps resulting from monomorphism between the mapping population parents. The old markers (prefixing with MS) integrated into the new version of the SSR physical map were re-prefixed with SM, and the new ones further post fixed with A and B, if any. The *Avr* genes isolated were also included in the SSR physical map with their sequence information. The methods used to identify SSRs, to design primers and to deploy the PCR assays followed those described by Feng et al. [15].

### Gene analysis

A monogenic line with resistance gene *Pi12*, IRBL12-M, the universal susceptible cultivar Lijiangxintuanheigu (LTH; also the recipient for a set of monogenic lines;), and other 10 monogenic cultivars/lines with the respective resistance genes, *Pia*, *Pii*, *Pik*, *Pik-p*, *Pit*, *Pi2*, *Pi11*, *Pib*, *Pita*, *Pita-2* [26] (data will be shown elsewhere), were used as the host cultivars in this study. Seeds of 12 cultivars were separately sown in a plastic pot (diameter 17 cm, height 9.5 cm). Three to five plants per cultivar were used for inoculation. The methods used for *Mo* inoculation and disease scoring followed those described elsewhere [27]. Each isolate was tested at least twice, and the highest disease score recorded was adopted as the true score. The pattern of segregation for *Avr* (disease rating of 0–2) to *Pi12* across the mapping population was expected to be 1:1 since *Mo* is haploid [10, 24, 25], and the observed ratio was tested against this expectation using a standard χ^2^ test.

### Linkage mapping

Initially, the bulked segregant analysis (BSA) [28] approach was taken to identify the genetic location of *AvrPi12.* The two necessary contrasting bulk DNAs were formed by creating an equimolar mixture of DNA extracted from either ten isolates which were all avirulent or ten which were all virulent when inoculated on IRBL12-M. The two bulk DNAs were tested with 132 genomically well dispersed SSR assays. SSRs identified as being potentially linked to *AvrPi12* according to the BSA assay were then used to genotype individual members of the mapping population. To refine the map location, this step was repeated using a number of newly developed PCR-based markers (single nucleotide polymorphisms and indels) known to lie within the critical genomic region. Since *Mo* is a haploid organism, the recombination frequency between adjacent markers is given by the ratio between the number of recombinants and the total number of individuals tested [10, 15, 24]. As the relevant recombination frequencies were all below 4.5%, they were directly equivalent to cM [24]. Since the markers used for mapping had been placed by inspection of the genome sequence of *Mo* isolate 70–15, it was possible to convert the genetic map into a physical one. The disordered genomic region between the donor isolate and the reference isolate 70–15, if any, was adjusted by the actual recombinants detected at the respective loci. The methods used to extract DNA from the *Mo* isolates, to develop the new markers and to perform genotyping followed those given by Ma et al. [24].

## Results

### The updated SSR-based *Mo* physical map

**Fig. 1:**
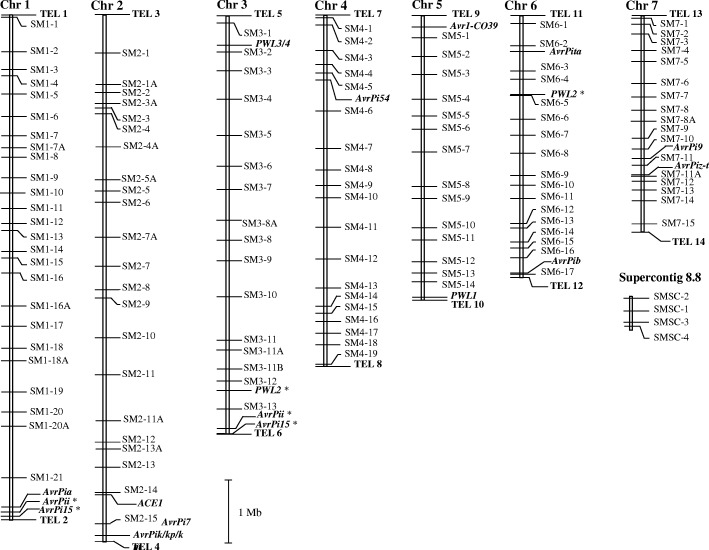
Updating the *Mo* SSR-based linkage map, based on the current version of the reference genome sequence of isolate 70–15 (MG8; https://www.ncbi.nlm.nih.gov/assembly/GCF_000002495.2/). The map was constructed using 116 of the 120 markers reported in [15], along with 18 newly developed ones (see also Additional file 1: Table S1). The telomeres were indicated by boldface, and the cloned *Avr* genes were integrated into the map with their sequences. *PWL1*, and *PWL3/4* [35], *PWL2* [36], *AvrPita* [31], *ACE1* [37], *AvrPiz-t* [43], *AvrPia* [33, 38], *AvrPii* and *AvrPik/kp/km* [33], *AvrPi54* [44], *AvrPi15* [24], *AvrPi7* [25]. *AvrPi15*, *AvrPii*, *Avr1-CO39* and *PWL2* located on double positions were marked by *. *AvrPia*, *AvrPii* and *Avr1-CO39*, which were absent on 70–15, were landed by their flanking sequences

Of the 120 SSR markers used to develop the original linkage map, 116 were retained in the updated version (Fig. 1; Additional file 1: Table S1; also see [15]). The four old markers, MS4–6, 4–7, 6–15, and 7–2, were ruled out, as their positions were missing in the MG8 (MS4–6, 7–2), or their assays proved to be non-robust (MS4–7, 6–15). A further 18 SSR loci were added to the set of 116. A comparison between the two versions of the physical map showed that just two of chromosomes (3 and 7) were almost maintained with respect to their SSR content, although their marker order was altered in both cases (see SM and MS marker codes in Additional file 1: Table S1). The marker content of the updated version of chromosome 1 was almost the same as that of the previous version of chromosome 2, and similarly for the new chromosome 5/previous chromosome 6 and the new chromosome 6/previous chromosome 4. The updated version of the chromosome 2 map incorporated SSRs previously allocated to chromosomes 1 and 4; while the new version of chromosome 4 combined markers previously associated with chromosomes 1, 3 and 5. An additional chromosome, referred to here as “Supercontig 8.8”, harbored markers previously assigned to chromosome 7. Thus the updated version of the linkage map comprised eight chromosomes, according to the MG8 genome (Fig. 1).

### Avirulence inheritance

The parental isolates CHL42 and CHL357 were, respectively, avirulent and virulent on IRBL12-M, while they were both virulent on LTH (Additional file 2: Figure S1). When the 219 progeny isolates were tested individually for their avirulence/virulence on IRBL12-M, avirulence and virulence segregated in a 1:1 ratio (111:108, χ^2^ = 0.02, *P* < 0.10). This result further suggested that the progeny population tested consisted of random ascospore isolates. The progeny population was, therefore, recognized as an appropriate mapping population for *AvrPi12*, which is responsible to the resistance gene *Pi12* carried by IRBL12-M.

### Chromosome landing

**Fig. 2:**
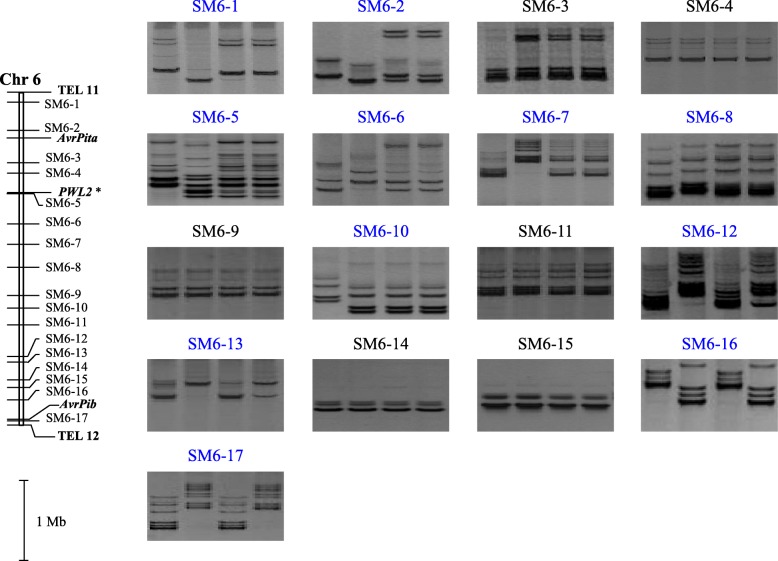
PCR profiles of chromosome 6 markers putatively linked to *AvrPi12* as a result of applying the bulked segregant analysis assay (also see Additional file 2: Figure S1). In each panel, lanes #1 through #4 represent, respectively, the isolate CHL42 (avirulent against *Pi12*), the isolate CHL357 (virulent), a bulk formed by ten avirulent progeny isolates and a bulk formed by ten virulent progeny isolates. Informative markers are shown in blue. *One of two possible genomic locations for *PWL2* [36] was suggested

**Fig. 3:**
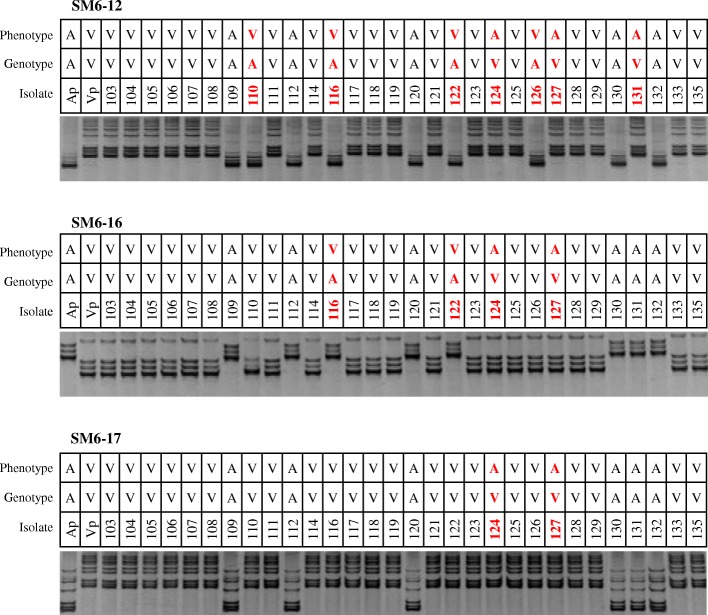
PCR profiles of 30 progeny isolates and their parental isolates derived from three linked markers. Phenotypes: A, avirulent; V, virulent. Genotypes: A, the same with A parent (Ap); V, the same with V parent (Vp). The recombinants were in red

To search for the chromosomal location for the *AvrPi12* locus, a total of 132 SSR markers covering the whole genome of *Mo*, were screened by BSA assay, of which 90 showed a polymorphism between two parental isolates (Fig. 2 and Additional file 3: Figure. S2). The results showed that markers on chromosomes 1–5 and 7 as well as Supercontig 8.8 were unlikely as candidate ones linked to the locus (Additional file 3: Figure. S2). In contrast, a series of markers located on chromosome 6 showed concurrent polymorphisms for both parental and bulk pairs (Fig. 2). Three polymorphic markers, SM6–12, SM6–16, and SM6–17, were selected for tentative validation with the progeny isolates ranged from #103 to #135. The results showed that all the three markers were linked to the *AvrPi12* locus (Fig. 3). Taking into a consideration that the *AvrPi12* locus might be rearranged by some segments from other chromosomes [10], four putative polymorphic markers, SM2–1, SM2–15, SM4–2, and SM7–14, were tested with the progeny isolates ranged from #1 to #035, individually. The results showed that all the four markers were not linked to the *AvrPi12* locus (Additional file 4: Figure. S3). Taken together, the data supported a location for *AvrPi12* on chromosome 6.

### Gene location

**Fig. 4:**
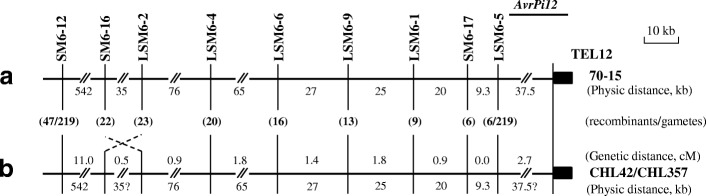
Genetic and physical maps of the *AvrPi12* locus. **a** A physical map of markers used for chromosome walking to *AvrPi12* locus based on the reference genome of isolate 70–15. **b** Genetic and physical map of the *AvrPi12* locus. The numbers shown below the map indicate distance between adjacent markers. Recombinants detected at each marker was shown in parenthesis, and the respective genetic distance between adjacent markers was shown above the map in cM (not shown to scale). The physical distances in the parental genomes of both isolates CHL42 and CHL357 were generally referred to those of 70–15, where the physical distances of two intervals were questioned, one was in an inversion between markers SM6–16 and LSM6–2, and another in the telomeric region between markers LSM6–5 and TEL12

Three above-mentioned markers were further tested with the rest progeny isolates in the mapping population. The results showed that 47, 22, and 6 recombinants, respectively, detected at markers SM6–12, SM6–16, and SM6–17 in the first round of linkage analysis (Fig. 4). Because all the recombinants from SM6–12, through SM6–16, and to SM6–17 overlapped, and the last one is close to the telomere of *Mo*, TEL12, the *AvrPi12* locus was defined between SM6–17 and TEL12 (Fig. 4). To construct a fine map of the locus, six additional markers were de novo developed for the second round of linkage analysis (Table 1). The results showed that there were 20, 16, 13, and 9 recombinants, detected at markers, LSM6–4, LSM6–6, LSM6–9, and LSM6–1, respectively, indicating that these markers were located to an interval between markers SM6–16 and SM6–17. As to marker LSM6–2, there were 23 recombinants, suggesting a genetic inversion between markers SM6–16 (22 recombinants) and LSM6–2 was occurring in the parental genome (Fig. 4). The six recombinants detected between *AvrPi12* and LSM6–5 were the same as those detected between *AvrPi12* and SM6–17, thereby narrowing the genetic window harboring *AvrPi12* to the interval between LSM6–5 and TEL12. Each genetic distance was determined by recombinants occurred in the region between adjacent markers, which was shown above the map in cM. The estimated physical distances between the various linked markers were mostly derived from the isolate 70–15 genome sequence; the exceptions involved the inferred inversion between SM6–16 and LSM6–2, and the interval between LSM6–5 and TEL12, which is of uncertain length, given that telomeric regions of many plant pathogens appear to be hypervariable [24, 29, 30]. As per the reference genomic sequence of 70–15, in this region, there were 12 candidate genes predicted for *AvrPi12* (Additional file 5: Table S2). Intriguingly, there was not any one meeting criterions for both secretion and effector, indicating that *AvrPi12* might be located on a specific interval, which was absent in the genome of 70–15.

**Table 1:** PCR-based markers linked to the *AvrPi12* locus mapping to *Mo* chromosome 6

Marker ^a^	Type ^b^	Primer sequence (5′ → 3′) ^c^	Genomic position (bp) ^d^	Tm (°C) ^e^	Size (bp) ^f^
SM6–12	SSR	F: CGTATTCTTGGCTGAGTGGC	6: 3283269	60	293
		R: GCCGACGACCTGTGTGATAC			
LSM6–2	InDel	F: GCGAGAGTTTGACTGATGTTTG	6: 3857026	60	86
		R: ATCCACCCAAGCTTTCGTTTTAG			
SM6–16	SSR	F:TGATACTAACTCCTCCTCCCAAAAC	6: 3826452	57	159
		R: CAGTCACGGTCTCCTAAGCC			
LSM6–4	InDel	F: GCAGTAGGTGAATTGTCTCGGT	6: 3932760	58	124
		R: AGTCACCCCTACTCTGTTGTTGT			
LSM6–6	SNP	F: ACCGAGTTTAGTGTTTTGGATGA	6: 4011510	58	165
		R: TCGAAGGTTTATGGTGCCAAT			
LSM6–9	SNP	F: CCACCCTGTGTCGTACTTCAATT	6: 4040268	58	112
		R: AAGATTGGTGGCCTGTCGTT			
LSM6–1	InDel	F: ATACCAGAACAAATTGCAAACAGC	6: 4065773	60	46
		R: GTTACACCGAGGAACTTGCTTG			
SM6–17	SSR	F: GGCGGCAAGTCGCTGAAGG	6: 4086125	58	330
		R: GAGTTTGAGACCTTGCGATT			
LSM6–5	SSR	F: GAGACGATGGGCCTCTAGCA	6: 4096513	59	143
		R: CTCCACGACGGTATGTTTGC			

^a^SM markers were the basic markers as shown in Fig. 1 and Additional file 1: Table S1, and LSM markers were de novo developed markers specific for the *AvrPi12* locus

^b^SSR, simple sequence repeat; SNP, single nucleotide polymorphism; InDel, insertion-deletion

^c^F, forward; R, reverse

^d^Genomic positions were based on the latest version of the reference genome sequence of isolate 70–15 (MG8; https://www.ncbi.nlm.nih.gov/assembly/GCF_000002495.2/)

^e^All runs began with one cycle at 94°C for 3 min, followed by 30 cycles at 94°C for 30 s, 55–62°C (annealing temperature, Tm) for 30 s, and 72°C for 1–1.5 min; with a final extension at 72°C for 7 min

^f^Amplicons obtained in the mapping population were separated by electrophoresis on 8% or 10% polyacrylamide gels

## Discussion

### The updated *Mo* physical map can serve as a platform for gene mapping and cloning

The first whole genome sequence of *Mo* was released by IRGBC in 2003 (MG5; [16]). Since then, a critical effort has been made by the IRGBC in collaboration with the Broad Institute, USA, to improve the accuracy and integrity of the reference sequence that led to the release of the latest version of the reference genome sequence (MG8; [17, 18]). Comparison of the two versions of the SSR physical maps established based on MG5 and MG8 revealed that there were certain changes in both the content and the order of each chromosomal marker set (Fig. 1; Additional file 1: Table S1; also see [15]). Such dramatic changes between the two versions of the SSR physical maps that, in turn, indicated that the new one would serve as a more robust platform for gene mapping and cloning. It is, however, necessary to take into account a careful consideration for the specificities of the pathogen genomes, such as variability and plasticity, when adopted any reference sequence and linkage/physical map for individual studies [10, 14, 25, 31–34]. As to the linkage/physical maps of the *AvrPi12* locus, there were two genomic intervals being evaluted in the current study, one for genetic inversion between markers SM6–16 and LSM6–2, and another for the telomeric region between LSM6–5 and TEL12. A close understanding of the dynamics of genomic structure surrounding the target locus is crucial for chromosome walking to the target region [10, 14, 25, 32, 34].

### The physical maps of *AvrPi12* can serve as a start point for deciphering molecular mechanism underlying durable resistance of Moroberekan

In the updated SSR physical map, some 14 *Avr* gene loci including three *PWL* loci, where some target genes have been isolated, have been mapped based on their sequence information (Fig. 1). Ten loci were located in the sub-telomeric and telomeric regions: *PWL1*, and *PWL3/4* [35], *PWL2* [36], *AvrPita* [31], *ACE1* [37], *AvrPia* [33, 38], *AvrPii* [33], *AvrPik/kp/km* [33], *Avr1-CO39* [39], and *AvrPib* [10]. A similar situation was also identified in other pathosystems [29, 30, 40]. It was well recognized that the allocation of *Avr* genes in the dynamic telomeric regions was indeed one of the most forceful strategies for the pathogens to confront resistance gene-driven positive selection [7, 10, 30, 31, 41]. As a result, it is in turn a bigger challenge for chromosome walking to the target locus in the hypervariable region [10, 24, 25, 41, 42]. However, the substantial genomic sequence resources developed for species belonging to the Magnaporthaceae family can facilitate an in silico reconstruction of the telomeric region of a given isolate (www.ncbi.nlm.nih.gov/assembly/organism/318829/latest/; [10, 24, 25]).

The blast resistance of Moroberekan appears to have help up very well despite its cultivation over many years across a large area of West Africa [19]. Its durability is thought to be due its harboring of the three major genes *Pi12*, *Pi5* and *Pi7*, in combination with an unknown number of minor genes [19, 20]. Consideration of its resistance spectra has concluded that the key determinant of its high level of blast resistance across Africa has been *Pi12*, while its interaction with the other two major genes may be as important in East Asia [21–23]. Because *AvrPi5* and *AvrPi7* are known to represent alleles of, respectively, *AvrPii* and *AvrPik* (unpublished data), the isolation of the *AvrPi12* should allow for studies on the gene-for-gene network underlying the durable resistance of Moroberakan. Based on the genetic and physical maps of the *AvrPi12* locus reported here, the next step forward will be to initiate a chromosome walk from LSM6–5 to TEL12.

## Conclusions

The current version of the *Mo* genome sequence has been exploited to update its SSR-based physical map. A comparison between the updated and the original versions of the physical maps has revealed alterations with respect to both marker content and marker order within most of the chromosomes. *AvrPi12* was mapped to chromosome 6 using a population of 219 progeny isolates derived from the cross CHL42 x CHL357. More detailed mapping showed that the gene lays in a telomeric region distal to the SSR locus LSM6–5; the latter marker could serve as the starting point for a chromosome walk to the target locus.

## Additional files

Additional file 1: **Table S1** Primer pair sequences, SSR motifs, genomic positions and PCR conditions of the 134 SSR markers used to construct the linkage map (DOCX 51 kb)

Additional file 2: **Figure S1** The distinct reactions derived from the parental isolates each interacted with the monogenic line carrying *Pi12*, IRBL12-M, and its susceptible recipient, LTH. (PDF 3420 kb)

Additional file 3: **Figure S2** PCR profiles of the first 30 progeny isolates and their parental isolates which derived from four putative polymorphic markers those selected from BSA analysis. (PPTX 199 kb)

Additional file 4: **Figure S3** Bulked segregant analysis PCR profiles for the 117 SSR markers mapping to the non-critical chromosomes 1–5, 7 and Supercontig 8.8 (17 markers on chromosome 6 were shown in Fig. 2). (PPTX 1322 kb)

Additional file 5: **Table S2** Candidate genes for *AvrPi12* that were predicted in the target region flanked by ZSM6 and TEL12 (DOCX 22 kb)

## Abbreviations

*Avr*: Avirulence
BSA: Bulked segregant assay
InDel: Insertion-deletion
IRBGP: International Rice Blast Genome Project
LTH: Lijiangxintuanheigu
*Mo*: *Magnaporthe oryzae*
SNP: Single nucleotide polymorphism
SSR: Simple sequence repeats
