# Supplementary material for: Reconstruction of an SSR-based Magnaporthe oryzae physical map to locate avirulence gene AvrPi12
Source: BMC Microbiol. 2018 May 31;18:47. doi: 10.1186/s12866-018-1192-x (PMC5984427; doi:10.1186/s12866-018-1192-x)
Supplement: Supplementary file 1 — Table S1 Primer pair sequences, SSR motifs, genomic positions and PCR conditions of the 134 SSR markers used to construct the linkage map (DOCX 51 kb) [file 12866_2018_1192_MOESM1_ESM.docx]

**Additional file 1: Table S1** Primer pair sequences, SSR motifs, genomic positions and PCR conditions of the 134 SSR markers used to construct the linkage map

| Marker ^a^ | Forward primer | Reverse primer | SSR motif | Genomic  position ^b^ | Expected length ^c^ | Tm  (℃) ^d^ |
| --- | --- | --- | --- | --- | --- | --- |
| **Chromosome 1** |  |  |  |  |  |  |
| SM1-1 (MS2-1) | ggtcccagtggctgctccg | ccaagccgacgagttcatcc | (tgc)11 | 1:43361 | 274 | 57 |
| SM1-2 (MS2-2) | cgtcgtctttcggctttgtag | gctgctcgcttgcggatggc | (acc)8 | 1:584535 | 172 | 57 |
| SM1-3 (MS2-3) | tgctcaattgagtacgccat | tcgtcctgacgccatatgc | (ta)21 | 1:869268 | 116 | 58 |
| SM1-4 (MS2-4) | ctgaagcaggtttaagcga | taatgctaaccccactacagg | (tac)17 | 1:964128 | 141 | 60 |
| SM1-5 (MS2-5) | ctggacatgaccgtttatgctact | cgtactgcacgattagctggt | (ta)13 | 1:1255656 | 128 | 56 |
| SM1-6 (MS2-6) | cggtgagtgagtattctgt | ggtcggcaggctccagattt | (tg)7(gc)7 | 1:1609091 | 98 | 60 |
| SM1-7 (MS2-8) | ttcctttccatcctcgtg | tctgattgtctcgtctgttg | (ga)14 | 1:1917040 | 169 | 56 |
| SM1-7A | tgtccgcaaaagcgagcaat | cttgggactagagataaaacgatgtg | (tg)10 | 1:2096316 | 77 | 54 |
| SM1-8 (MS2-9) | ggtcagcagcaatcgcgaac | aaatcaggaatcgatccgcag | (tg)20 | 1:2252903 | 90 | 60 |
| SM1-9 (MS2-10) | tcctaattctctttcttactctctc | gataataaccggttcaaggc | (ct)12 | 1:2577769 | 74 | 56 |
| SM1-10 (MS7-17) | tagacttagatagcgcgccgt | gtctctcgggtgcggattta | (tac)34 | 1:2817542 | 338 | 60 |
| SM1-11 (MS2-11) | ggcgacttggcattggatt | aacgttacctacagtttcccagc | (ttc)12 | 1:3063137 | 118 | 60 |
| SM1-12 (MS2-12) | gacctgcaacgaccaaattctc | aatcgaataggttgaaacatgctct | (at)29 | 1:3299791 | 126 | 60 |
| SM1-13 (MS2-13) | taagggaatgctaataccg | ttacttacttgcttacctgag | (at)21 | 1:3412267 | 255 | 56 |
| SM1-14 (MS2-14) | ttttctaccctttacgtgggatgt | taaaagtgatatcccctcgcaag | (gt)19 | 1:3747170 | 113 | 60 |
| SM1-15 (MS2-15) | ggagaagaacaatctggagg | gagcccttatcttcctgcc | (tc)21 | 1:3846224 | 243 | 60 |
| SM1-16 (MS2-16) | caggcaaggaaatgggcac | gatgaggcgggtatccaag | (taa)9 | 1:4082830 | 152 | 60 |
| SM1-16A | gtgaacagggtgctggcgt | aagccacccaagccatcagt | (gt)16 | 1:4764305 | 97 | 62 |
| SM1-17 (MS2-17) | cgcaaaccacgactcctccag | cggcacgacgacaacgaccc | (gct)8 | 1:4924947 | 155 | 60 |
| SM1-18 (MS2-18) | tttgctctcgggcgtggaaac | cgctttctctatcgctacttgc | (ct)9 | 1:5268306 | 168 | 60 |
| SM1-18A | caccacgtcctcgatgatctc | tgctaacggccggctaatg | (gta)8 | 1:5549748 | 124 | 60 |
| SM1-19 (MS2-19) | caaggacaagcgtaaatggc | gacagcagccgactttcct | (cag)10 | 1:5965963 | 131 | 60 |
| SM1-20 (MS2-20) | ccgaggacgacgacgagat | cagccagcgttgcttcgtcg | (cag)9 | 1:6279754 | 203 | 60 |
| SM1-20A | ggcctcatcttcagcgttatc | aggggatgaagatgaagatgaag | (ctt)13 | 1:6501423 | 100 | 60 |
| SM1-21 (MS4-4) | gccacaagcttgcctgttaac | ggcgtcagtacgtcagagtt | (ga)10 | 1: 7317739 | 205 | 56 |
| **Chromosome 2** |  |  |  |  |  |  |
| SM2-1 (MS4-3) | gtcagcgaggcaagaggtatg | gtacatgcacatctgagaccc | (tc)9 | 2:604893 | 184 | 53 |
| SM2-1A | aaggcgtcttggaaccatga | gtggattgtgcatgatcatgatttg | (ct)14 | 2:1083820 | 100 | 54 |
| SM2-2 (MS4-5) | cagtgatcacctcatggctc | cctgtagacgagccaaacgg | (agc)10 | 2:1227662 | 224 | 60 |
| SM2-3A | acctactttgcaaccttgataataac | gggtcgcctatgtacggat | (ca)14 | 2:1321940 | 77 | 52 |
| SM2-3 (MS4-8) | cctgggtacgtaggttctcac | ccacttgctggatggtttgc | (tacc)6 | 2:1489115 | 163 | 60 |
| SM2-4 (MS1-1) | gctgctgtgcctgcctttg | gaggcaacaacaacagcgaaat | (tgc)10 | 2:1570384 | 74 | 57 |
| SM2-4A | ttctgtcttctcttttagtctatatct | gtgatgggcactcatgttg | (ta)11 | 2:2110308 | 89 | 50 |
| SM2-5A | tggacgcaacaggtaataaaaaag | ttgtttatttccagtggctgtgta | (ga)11 | 2:2575862 | 76 | 52 |
| SM2-5 (MS1-2) | cgacgcatcataacagcctc | gcagaacattgccaactacg | (gct)10 | 2:2787442 | 305 | 60 |
| SM2-6 (MS1-3) | gctgtccaaccctcactgct | gttgttcgtcacacccttg | (ca)25 | 2:2965729 | 295 | 60 |
| SM2-7A | gaccaagttaccctgtcatac | acgagaaccccttgtgtgg | (gt)15 | 2:3460710 | 76 | 52 |
| SM2-7 (MS1-4) | ggaaggcagcgactctgaaag | cctgttgtcgtctccacttt | (atg)10 | 2:3977118 | 147 | 56 |
| SM2-8 (MS1-7) | cagtgcttggttcttgggag | ctaacctcccctgtcgctct | (tg)20 | 2:4344657 | 259 | 60 |
| SM2-9 (MS1-5) | tcgtagagtagtttccagtc | cgctcgaggatagtcgtgtt | (ta)22 | 2:4475019 | 186 | 60 |
| SM2-10 (MS1-6) | gactcccatagcggtgtttg | caagacctccacaccatccag | (cag)11 | 2:5103789 | 177 | 56 |
| SM2-11 (MS1-13) | tggtggaagcacgacggaat | gcttgtgctctcccgactc | (ct)11 | 2:5688063 | 224 | 56 |
| SM2-11A | cctgaagctggtgtcgtttgg | ccgaatgaactccaccgca | (gtt)18 | 2:6479532 | 94 | 58 |
| SM2-12 (MS1-8) | ttagaccgtcatcgccatcag | caacggaaatggtgaggtat | (ct)18 | 2:6755376 | 229 | 60 |
| SM2-13A | tatctcgtgcaggccggtgta | gcaggtgagcaaacagcaagac | (ct)48 | 2:6844629 | 214 | 58 |
| SM2-13 (MS1-9) | ccaagaaaccaagtccatcc | gactcctgtgttgattgcgg | (ac)19 | 2:7147592 | 256 | 56 |
| SM2-14 (MS1-14) | gttgctaacggccggctaa | ttgatcaaggcatactttcgca | (tac)14 | 2:7477685 | 115 | 57 |
| SM2-15 (MS1-15) | ctggactgctgggtggttc | caggcaagactatggcaagg | (ca)24 | 2:8042296 | 240 | 56 |
| **Chromosome 3** |  |  |  |  |  |  |
| SM3-1 (MS7-6) | gtttgacgtggatattcgcc | gcagcattcacatcttcagc | (ta)17 | 3:115558 | 531 | 60 |
| SM3-2 (MS3-1) | ctgttgctgctgttgcgac | tggcgggacggacaatagtg | (gtt)16 | 3:574533 | 204 | 60 |
| SM3-3 (MS3-2) | cctttcctccttacctacctctg | gggtttcggttagatatacgtgc | (tc)14 | 3:869090 | 79 | 54 |
| SM3-4 (MS3-3) | ggtctcggtgtggtagaaacg | gcagtagtggccgtcgacg | (tgtcgtcgt)10 | 3:1321165 | 150 | 58 |
| SM3-5 (MS3-9) | cgcatcctgtccgactgaaag | tggccgtcattgagtgcgtc | (tc)22 | 3:1889955 | 281 | 56 |
| SM3-6 (MS3-8) | catacttcttcttaccctcttattcct | tttcagatctctccgaggacaa | (tc)36 | 3:2379908 | 138 | 54 |
| SM3-7 (MS3-7) | tgccttgccgagaccaccg | gtccgaccctcacagcatc | (ct)14 | 3:2743023 | 266 | 60 |
| SM3-8A | ctgaattggtactcaaaggaa | cttttctgtctgtctgtcgttt | (ta)10 | 3:3269208 | 120 | 50 |
| SM3-8 (MS3-4) | ggagcagcagtctggagtc | ccttccagagtctttccgtag | (gt)15 | 3:3543670 | 78 | 54 |
| SM3-9 (MS3-18) | gcaaacatcatctgggag | catctactttactgccacc | (at)11 | 3:3869076 | 251 | 56 |
| SM3-10 (MS3-17) | ccagagagaagaagtgcgt | ggctggcacagaaccaaggc | (at)16 | 3:4438419 | 310 | 60 |
| SM3-11 (MS3-16) | tttttagcgggaggacttgtt | attcccatggtcctagtcgc | (tc)18 | 3:5131269 | 91 | 54 |
| SM3-11A | gactaaaaatgtgtagaagcctc | ctgtaactgttaggcatgaga | (ct)44 | 3:5467912 | 126 | 50 |
| SM3-11B | gtggccaaaaagtacaggtg | gactcagcccctccacttt | (ta)15 | 3:5495506 | 83 | 54 |
| SM3-12 (MS3-19) | caggacggctgcgaggagaac | gctgctgctgcgttgccgac | (tgc)12 | 3:5767542 | 140 | 56 |
| SM3-13 (MS3-20) | aatgaaaactaaaaagtaaacaac | tcaattaagggacggaggc | (ac)15 | 3:6101011 | 75 | 56 |
| **Chromosome 4** |  |  |  |  |  |  |
| SM4-1 (MS5-10) | gaccaggctgtctctcccgt | cgggagagactgagatggac | (ctg)10 | 4:38406 | 204 | 60 |
| SM4-2 (MS5-9) | ccacgtagtactcgacgagc | ctggagggtcctacagcatc | (ttg)11 | 4:155020 | 254 | 60 |
| SM4-3 (MS5-8) | gacgacgacgcagacaaactt | atgatggcggtcgagaagat | (cag)10 | 4:557822 | 99 | 56 |
| SM4-4 (MS5-7) | gccgtcgatccagttacaac | gcggactagcttgactggatg | (ta)22 | 4:778195 | 348 | 56 |
| SM4-5 (MS3-15) | cgccaataactccagcagcc | tagattagtgttagtaaagac | (at)15 | 4:915998 | 366 | 60 |
| SM4-6 (MS3-14) | caatgtatttccacggtagt | ggtgtccgttcttgtatgat | (ca)16 | 4:1512880 | 214 | 60 |
| SM4-7 (MS3-13) | gatgccaacttatcgggtcg | tgcttccgccactcaactt | (aat)81 | 4:2106055 | 404 | 60 |
| SM4-8 (MS3-12) | ctcccaagcaagcgatgcc | gactaaaggaagggaggag | (tg)17 | 4:2445091 | 269 | 60 |
| SM4-9 (MS3-11) | gtcaaatctgtttttccccct | ggtcaggaacaaccagcac | (tc)19 | 4:2689701 | 79 | 52 |
| SM4-10 (MS3-10) | cctgttttctatgtacctatcga | ggagtacatgcaagcgctt | (ga)14 | 4:2883888 | 88 | 50 |
| SM4-11 (MS1-12) | cccacatacaaacacatacgt | catttttggtggcgttgag | (ac)16 | 4:3349556 | 77 | 52 |
| SM4-12 (MS1-11) | ggtcacttggtcaaggttctag | gctggggactgcctacctt | (tacc)14 | 4:3850527 | 131 | 56 |
| SM4-13 (MS1-10) | cgcctctcttggtattgg | gtccttgatgaacaaacact | (cttgc)29 | 4:4308609 | 358 | 60 |
| SM4-14 (MS5-6) | cttgggagccatggtggttc | caaaggcgcaccaagaccatg | (gt)13 | 4:4599427 | 268 | 60 |
| SM4-15 (MS5-5) | tttggagcggaccttggatg | actcaagaaactgatcgacggct | (gaa)12 | 4:4708312 | 136 | 58 |
| SM4-16 (MS5-4) | agagcagccagggttaggagtac | gagcttcttcaatgctgcgg | (cca)9 | 4:4838799 | 133 | 58 |
| SM4-17 (MS5-3) | gctgcaaggtcgacaaggg | cgcagcatcgctaccacca | (ggc)8 | 4:5023751 | 68 | 60 |
| SM4-18 (MS5-2) | ctgagcttgaggaggtcgtcg | gagcgagtatcccaagaagg | (at)16 | 4:5205542 | 244 | 56 |
| SM4-19 (MS5-1) | cccattatctcctcgtcag | caaccactgccgactgatac | (caaa)43 | 4:5514753 | 385 | 60 |
| **Chromosome 5** |  |  |  |  |  |  |
| SM5-1 (MS6-1) | caccaggtgtaacttgagg | cctaaaggaatccagcatc | (gt)14 | 5:351510 | 75 | 50 |
| SM5-2 (MS6-2) | taccgacagggaacccagaac | atcctgctggggtccggtt | (cag)11 | 5:647144 | 80 | 60 |
| SM5-3 (MS6-3) | ccagtcggttccaggtcttg | ccacatcgcagcctcacttg | (gca)10 | 5:929398 | 197 | 60 |
| SM5-4 (MS6-4) | cattcccccagtggcggt | tagaaatgatacgagcaggccac | (aggt)12 | 5:1319597 | 154 | 56 |
| SM5-5 (MS6-5) | aagtgtgcagtaatatttctagg | tgatggccaggtcagcttt | (aaaaaaaaag)10 | 5:1584855 | 158 | 52 |
| SM5-6 (MS6-6) | catgaaggttttgttggtgcg | gacgaccgaatcttcttgcatat | (gt)11 | 5:1793594 | 78 | 56 |
| SM5-7 (MS6-7) | tcagcgtcagtctagcacaatg | cgcgccaccagtcataaca | (tg)24 | 5:2154572 | 90 | 56 |
| SM5-8 (MS6-8) | aacgcccaggtgagtcctaatt | agactgactatttttgctccgaga | (ta)21 | 5:2699012 | 149 | 56 |
| SM5-9 (MS6-9) | gtagggataactcctcagc | cgcgctgcgatgaggtact | (ac)10 | 5:2891211 | 190 | 53 |
| SM5-10 (MS6-10) | tttgcatgtatccttcctccgt | aaataacaaagatgccgaaaaagg | (tca)11 | 5:3347757 | 80 | 52 |
| SM5-11 (MS6-11) | ggtcgcgtcagtgaacaaac | cccgttgctcctcaatcag | (tac)8 | 5:3544298 | 295 | 60 |
| SM5-12 (MS6-12) | atagcatccatctccccaaca | tgtatgtaaggcgatgttgagaa | (caa)9 | 5:3890674 | 73 | 54 |
| SM5-13 (MS6-13) | gtacatgactgccaggcgtg | gacaggccagccacacttg | (aggt)8 | 5:4064284 | 206 | 60 |
| SM5-14 (MS6-14) | accatgcagacgagaccagaa | aagaagccgcgcaaggac | (ga)17 | 5:4179921 | 402 | 58 |
| **Chromosome 6** |  |  |  |  |  |  |
| SM6-1 (MS4-2) | gctggactgtggcattctg | cagggtcactaggcagagg | (cagcaaa)14 | 6:129727 | 333 | 60 |
| SM6-2 (MS4-1) | tgcgtcgctggatgggtct | cttcagaaacatctccccaccaa | (gtt)9 | 6:459241 | 113 | 58 |
| SM6-3 (MS4-9) | ggcagtgatgtcgtccaaag | gcggtcgaggagaaacaatc | (ga)9 | 6:874744 | 230 | 52 |
| SM6-4 (MS4-10) | gacaggtacgatgtgcttgc | ctgttgcctgctttcccatc | (tagg)9 | 6:1006333 | 238 | 60 |
| SM6-5 (MS4-17) | tctttaattaatatatttgcatccat | gcccggcgttaaataatact | (agt)8 | 6:1246720 | 70 | 45 |
| SM6-6 (MS4-16) | gctgttgatgttgctggg | gccagtcctaacacgatg | (gtt)11 | 6:1633892 | 195 | 56 |
| SM6-7 (MS4-15) | ggaacgtgtacgtggtcaac | ggcactcaaggcttctctcg | (ct)16 | 6:1889041 | 242 | 60 |
| SM6-8 (MS4-14) | tggtacacacagtagacgccgtt | caccgctaaaataaattcccgtc | (ct)33 | 6:2177428 | 136 | 54 |
| SM6-9 (MS4-13) | gtttgcctgtagggtggtag | ccgagtctgaacttctggg | (gt)10 | 6:2553634 | 193 | 60 |
| SM6-10 (MS4-12) | taaagttaggtatgcaatattcgtg | ggtagagtttacgtacatagacacac | (gt)24 | 6:2679791 | 104 | 50 |
| SM6-11 (MS4-11) | gcgggttctcgtagatgtc | gacagcgccatgaaggaga | (ct)24 | 6:2890315 | 263 | 60 |
| SM6-12 (MS4-18) | cgtattcttggctgagtggc | gccgacgacctgtgtgatac | (ga)14 | 6: 3283269 | 293 | 60 |
| SM6-13 (MS4-19) | cgtactccgtacgtacactc | gcgctggagctatcgagttc | (gtaaggca)15 | 6:3360982 | 269 | 58 |
| SM6-14 (MS4-20) | tgcgcttctacaacaaccag | ctcggaaaggaccgtctcg | (cgt)6 | 6:3576091 | 92 | 56 |
| SM6-15 (MS4-21) | tggaacgagccaaaggaagc | cgagcccggcgttaactaata | (agt)8 | 6:3669049 | 70 | 58 |
| SM6-16 (MS3-6) | tgatactaactcctcctcccaaaac | cagtcacggtctcctaagcc | (ga)14 | 6: 3826452 | 159 | 57 |
| SM6-17 (MS3-5) | ggcggcaagtcgctgaagg | gagtttgagaccttgcgatt | (tc)41 | 6:4086125 | 330 | 58 |
| **Chromosome 7** |  |  |  |  |  |  |
| SM7-1 (MS7-20) | ccacaacgcgattacgaggt | gacgagacgaaagctgccat | (caccacct)10 | 7:42746 | 358 | 60 |
| SM7-2 (MS7-19) | ccatcggcaatgatactgtg | cagggtcgttcagggtcttc | (ca)17 | 7:142073 | 201 | 60 |
| SM7-3 (MS7-16) | gagtgctgctgctggtgctt | cccgggacaatagcaacagc | (tgt)32 | 7:293667 | 249 | 60 |
| SM7-4 (MS7-15) | gaactgcgtacggactaggt | gggtgggaaggacaagacg | (gt)13 | 7:555822 | 183 | 60 |
| SM7-5 (MS7-14) | ggtcagcgtattgggagcag | ctgtaacggatcgacctgtc | (gt)11 | 7:731561 | 288 | 62 |
| SM7-6 (MS7-13) | cgactggatgcgctcgatag | catggtcctcactatcctgg | (ta)23 | 7:1073856 | 226 | 60 |
| SM7-7 (MS7-12) | cccaatcctcaacttctccc | cggttggtgaggaacagtgc | (tc)18 | 7:1288230 | 189 | 60 |
| SM7-8 (MS7-11) | tgcgcacacttgtacagaa | cctcaaaaagctatgcgtc | (ctga)9 | 7:1624682 | 73 | 52 |
| SM7-8A | gaaagagacaaaacactggctac | atttgcgggcggacactta | (ag)30 | 7:1799558 | 110 | 54 |
| SM7-9 (MS7-10) | ggagtagctgagaaggaggg | ggcctcgatcattgatggac | (ct)9 | 7:1938496 | 214 | 60 |
| SM7-10 (MS7-9) | cctaatcttgtcagcctgcc | cctcctccttcacgatgtag | (tagg)25 | 7:2107843 | 243 | 60 |
| SM7-11 (MS7-8) | tggacgaggtcaggtggga | ttcacgtctcaccaaacataaacact | (ct)8 | 7:2366098 | 117 | 56 |
| SM7-11A | cgagcccggcgttaaataat | gcagtgcagtgccattggtaa | (tac)21 | 7:2546496 | 106 | 56 |
| SM7-12 (MS7-5) | ggtcgcgtcagtgaactaac | ggtcggcatctagctctgag | (agt)9 | 7:2620871 | 234 | 60 |
| SM7-13 (MS7-3) | gctcgattcgcttctatgcc | gaggctcagctagctttccg | (cta)7 | 7:2924763 | 285 | 60 |
| SM7-14 (MS7-4) | gcaagcaggagatgcagac | gtgagatggtgtgccgtttg | (ca)21 | 7:2753446 | 215 | 60 |
| SM7-15 (MS7-1) | acagtgtcttgtgtatgtattcg | cacgcacagacgtaaaatt | (ca)13 | 7:3290202 | 71 | 50 |
| **Supercontig 8.8** |  |  |  |  |  |  |
| SMSC-1 (MS7-18) | ggccatagttcacgctcctc | gccgatggctcttgggtttg | (tag)8 | 8.8: 246659 | 291 | 60 |
| SMSC-2 (MS7-7) | gtcgcaacaacaacctctg | ccatgtgggccatactac | (ttg)37 | 8.8: 42519 | 257 | 56 |
| SMSC-3 | gcagccactggtcccgaca | cgagcccggcgttaactact | (agt)10 | 8.8: 378524 | 64 | 60 |
| SMSC-4 | tagttctagattattcgtaagctt | ccgggcagttggggtagc | (ctac)18 | 8.8: 456316 | 120 | 50 |

^a^Marker codes with MS those in brackets were old ones, which were previouly developed based on the older version of the reference sequence of isolate 70-15 (MG5; <http://www.ncbi.nlm.nih.gov/genomeprj/13840>; [15, 16]), and those with SM were new ones reassembled based on the latest version of the reference genome sequence (MG8; <https://www.ncbi.nlm.nih.gov/assembly/GCF_000002495.2/>; [17, 18]), and the SM with A, B were *de novo* developed for equalization of the parantally polymorphic markers for BSA analysis of *AvrPi12* in the population.

^b^Genomic positions were reassemblyed based on the MG8.

^c^ Fragment sizes (bp) as estimated from 10.0% denaturing polyacrylamide gel electrophoresis.

^d^ PCR annealing temperature.
