# Supplementary material for: Reconstruction of an SSR-based Magnaporthe oryzae physical map to locate avirulence gene AvrPi12
Source: BMC Microbiol. 2018 May 31;18:47. doi: 10.1186/s12866-018-1192-x (PMC5984427; doi:10.1186/s12866-018-1192-x)
Supplement: Supplementary file 2 — Figure S1 The distinct reactions derived from the parental isolates each interacted with the monogenic line carrying Pi12, IRBL12-M, and its susceptible recipient, LTH. (PDF 3420 kb) [file 12866_2018_1192_MOESM2_ESM.pdf]

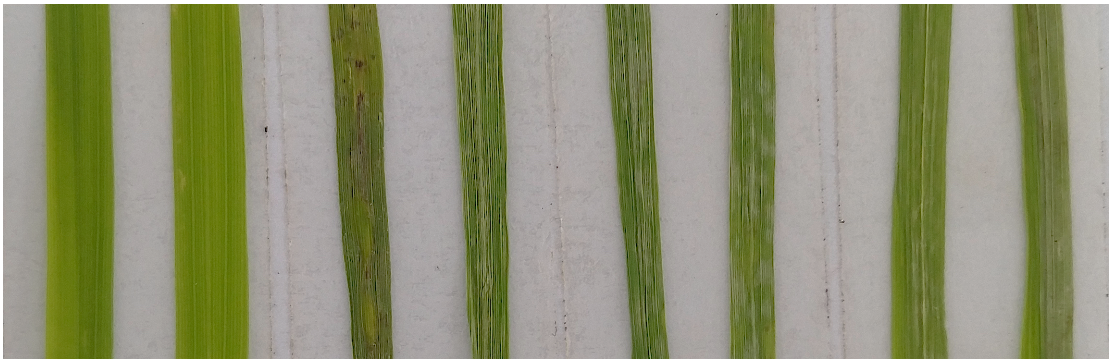

IRBL12-M LTH IRBL12-M LTH

CHL42 CHL357

Additional file 2: Fig. S1. The distinct reactions derived from the parental isolates each interacted with the monogenic line carrying *Pi12*, IRBL12-M, and its susceptible recipient, LTH.
