# Supplementary material for: Reconstruction of an SSR-based Magnaporthe oryzae physical map to locate avirulence gene AvrPi12
Source: BMC Microbiol. 2018 May 31;18:47. doi: 10.1186/s12866-018-1192-x (PMC5984427; doi:10.1186/s12866-018-1192-x)
Supplement: Supplementary file 3 — Figure S2 PCR profiles of the first 30 progeny isolates and their parental isolates which derived from four putative polymorphic markers those selected from BSA analysis. (PPTX 199 kb) [file 12866_2018_1192_MOESM3_ESM.pptx]

## Slide 1
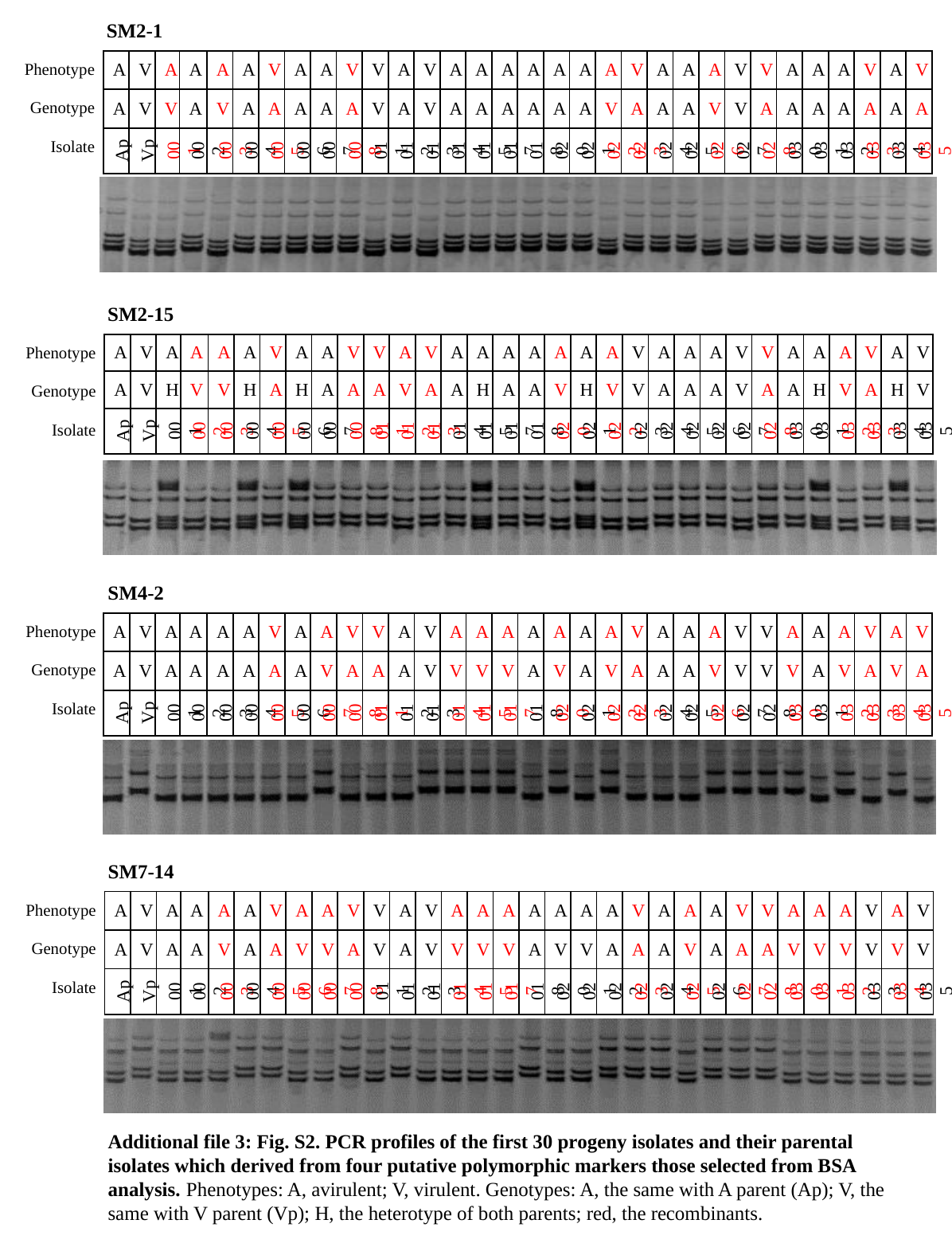

SM2-1
| A | V | A | A | A | A | V | A | A | V | V | A | V | A | A | A | A | A | A | A | V | A | A | A | V | V | A | A | A | V | A | V |
| --- | --- | --- | --- | --- | --- | --- | --- | --- | --- | --- | --- | --- | --- | --- | --- | --- | --- | --- | --- | --- | --- | --- | --- | --- | --- | --- | --- | --- | --- | --- | --- |
| A | V | V | A | V | A | A | A | A | A | V | A | V | A | A | A | A | A | A | V | A | A | A | V | V | A | A | A | A | A | A | A |
| Ap | Vp | 001 | 002 | 003 | 004 | 005 | 006 | 007 | 008 | 011 | 012 | 013 | 014 | 015 | 017 | 018 | 020 | 021 | 022 | 023 | 024 | 025 | 026 | 027 | 028 | 030 | 031 | 032 | 033 | 034 | 035 |
Phenotype
Genotype
Isolate
SM2-15
| A | V | A | A | A | A | V | A | A | V | V | A | V | A | A | A | A | A | A | A | V | A | A | A | V | V | A | A | A | V | A | V |
| --- | --- | --- | --- | --- | --- | --- | --- | --- | --- | --- | --- | --- | --- | --- | --- | --- | --- | --- | --- | --- | --- | --- | --- | --- | --- | --- | --- | --- | --- | --- | --- |
| A | V | H | V | V | H | A | H | A | A | A | V | A | A | H | A | A | V | H | V | V | A | A | A | V | A | A | H | V | A | H | V |
| Ap | Vp | 001 | 002 | 003 | 004 | 005 | 006 | 007 | 008 | 011 | 012 | 013 | 014 | 015 | 017 | 018 | 020 | 021 | 022 | 023 | 024 | 025 | 026 | 027 | 028 | 030 | 031 | 032 | 033 | 034 | 035 |
Phenotype
Genotype
Isolate
SM4-2
| A | V | A | A | A | A | V | A | A | V | V | A | V | A | A | A | A | A | A | A | V | A | A | A | V | V | A | A | A | V | A | V |
| --- | --- | --- | --- | --- | --- | --- | --- | --- | --- | --- | --- | --- | --- | --- | --- | --- | --- | --- | --- | --- | --- | --- | --- | --- | --- | --- | --- | --- | --- | --- | --- |
| A | V | A | A | A | A | A | A | V | A | A | A | V | V | V | V | A | V | A | V | A | A | A | V | V | V | V | A | V | A | V | A |
| Ap | Vp | 001 | 002 | 003 | 004 | 005 | 006 | 007 | 008 | 011 | 012 | 013 | 014 | 015 | 017 | 018 | 020 | 021 | 022 | 023 | 024 | 025 | 026 | 027 | 028 | 030 | 031 | 032 | 033 | 034 | 035 |
Phenotype
Genotype
Isolate
SM7-14
| A | V | A | A | A | A | V | A | A | V | V | A | V | A | A | A | A | A | A | A | V | A | A | A | V | V | A | A | A | V | A | V |
| --- | --- | --- | --- | --- | --- | --- | --- | --- | --- | --- | --- | --- | --- | --- | --- | --- | --- | --- | --- | --- | --- | --- | --- | --- | --- | --- | --- | --- | --- | --- | --- |
| A | V | A | A | V | A | A | V | V | A | V | A | V | V | V | V | A | V | V | A | A | A | V | A | A | A | V | V | V | V | V | V |
| Ap | Vp | 001 | 002 | 003 | 004 | 005 | 006 | 007 | 008 | 011 | 012 | 013 | 014 | 015 | 017 | 018 | 020 | 021 | 022 | 023 | 024 | 025 | 026 | 027 | 028 | 030 | 031 | 032 | 033 | 034 | 035 |
Phenotype
Genotype
Isolate
Additional file 3: Fig. S2. PCR profiles of the first 30 progeny isolates and their parental isolates which derived from four putative polymorphic markers those selected from BSA analysis. Phenotypes: A, avirulent; V, virulent. Genotypes: A, the same with A parent (Ap); V, the same with V parent (Vp); H, the heterotype of both parents; red, the recombinants.
