# Supplementary material for: Reconstruction of an SSR-based Magnaporthe oryzae physical map to locate avirulence gene AvrPi12
Source: BMC Microbiol. 2018 May 31;18:47. doi: 10.1186/s12866-018-1192-x (PMC5984427; doi:10.1186/s12866-018-1192-x)
Supplement: Supplementary file 4 — Figure S3 Bulked segregant analysis PCR profiles for the 117 SSR markers mapping to the non-critical chromosomes 1–5, 7 and Supercontig 8.8 (17 markers on chromosome 6 were shown in Fig. 2). (PPTX 1322 kb) [file 12866_2018_1192_MOESM4_ESM.pptx]

## Slide 1
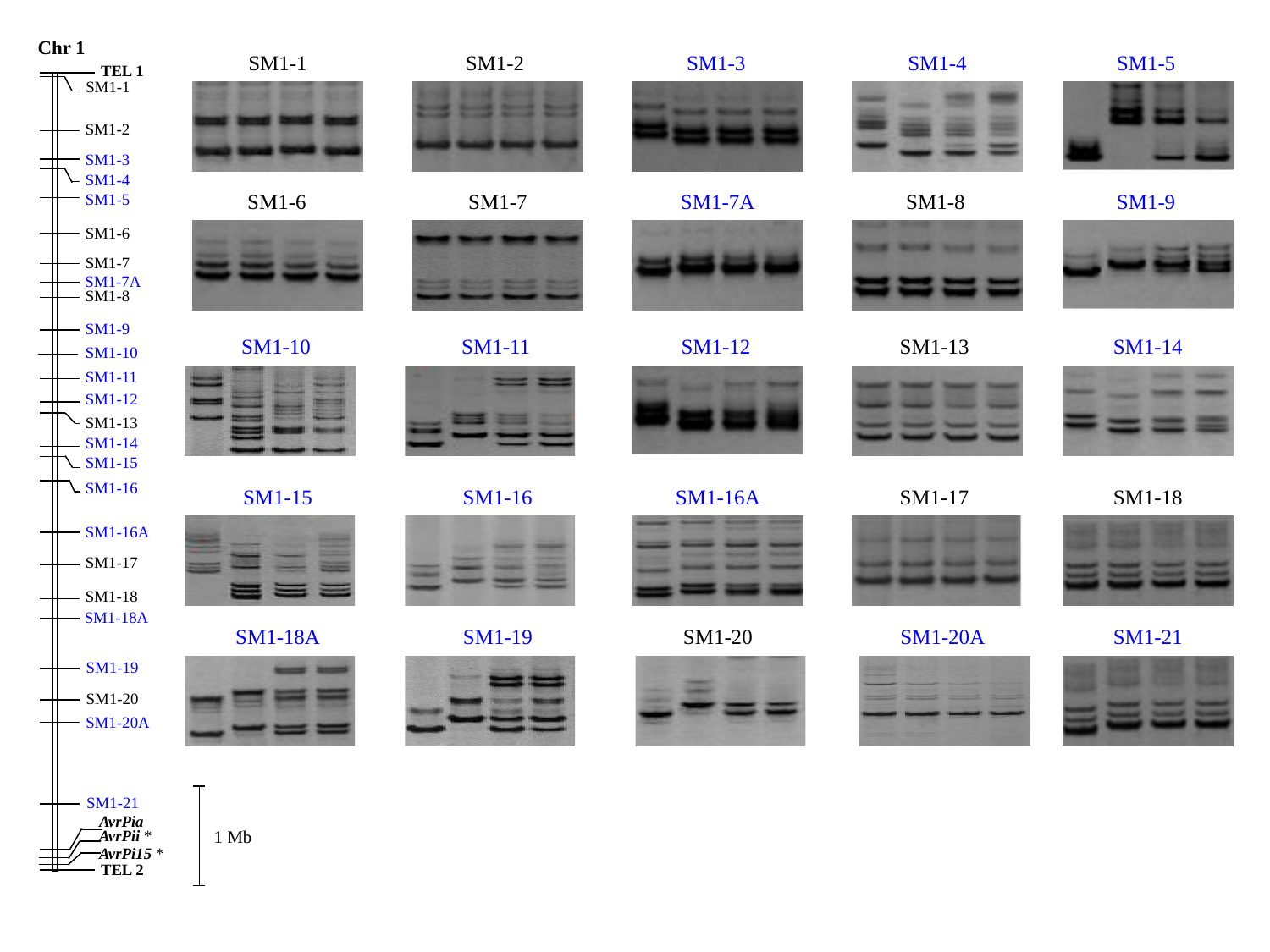

Chr 1
TEL 1
SM1-1
SM1-2
SM1-3
SM1-4
SM1-5
SM1-6
SM1-7
SM1-7A
SM1-8
SM1-9
SM1-10
SM1-11
SM1-12
SM1-13
SM1-14
SM1-15
SM1-16
SM1-16A
SM1-17
SM1-18
SM1-18A
SM1-19
SM1-20
SM1-20A
SM1-21
AvrPi15 *
TEL 2
AvrPia
AvrPii *
SM1-1
SM1-2
SM1-3
SM1-4
SM1-5
SM1-6
SM1-7
SM1-7A
SM1-8
SM1-9
SM1-10
SM1-11
SM1-12
SM1-13
SM1-14
SM1-15
SM1-16
SM1-16A
SM1-17
SM1-18
SM1-18A
SM1-19
SM1-20
SM1-20A
SM1-21
1 Mb

## Slide 2
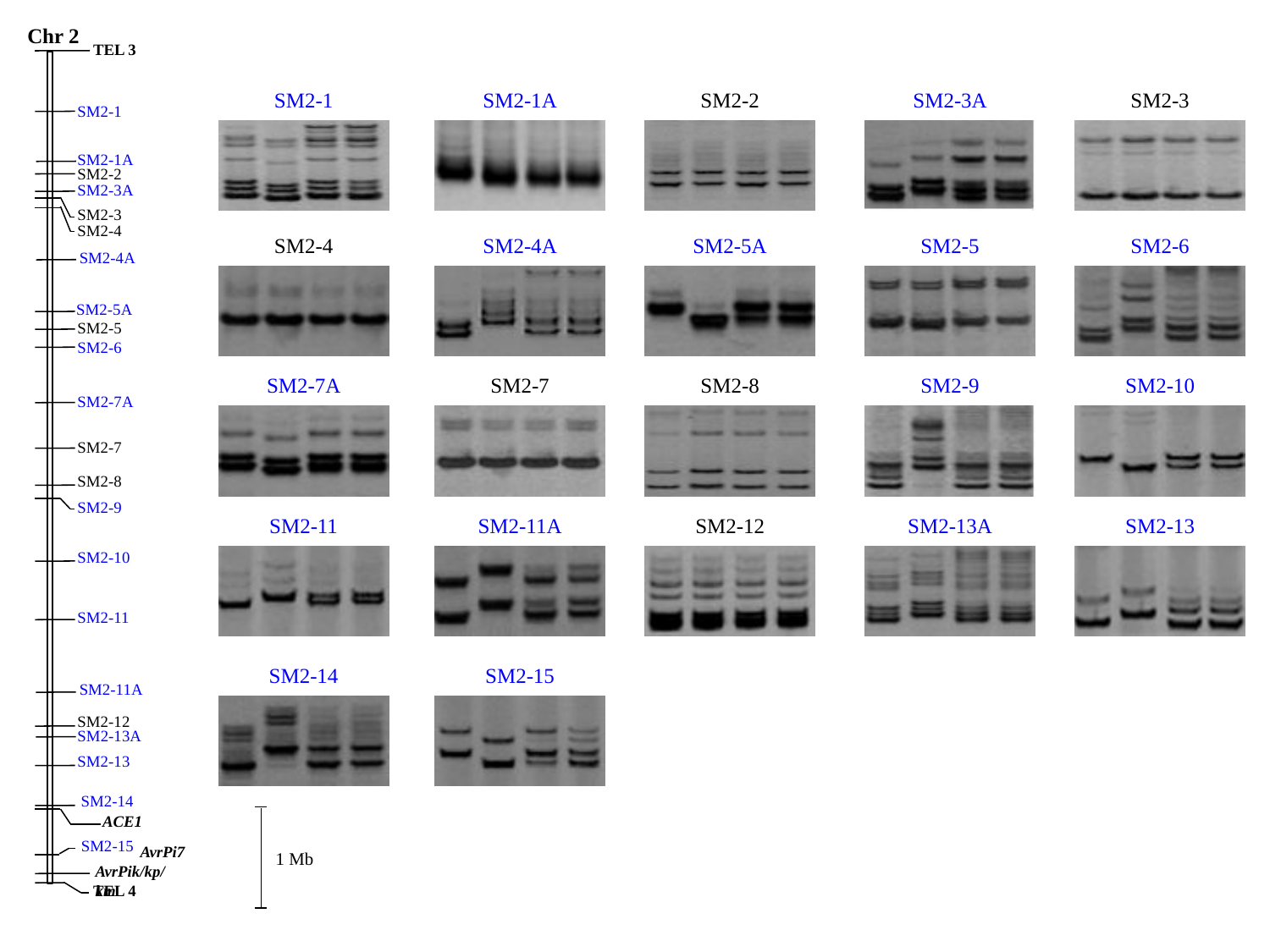

Chr 2
TEL 3
SM2-1
SM2-1A
SM2-2
SM2-3A
SM2-3
SM2-4
SM2-4A
SM2-5A
SM2-5
SM2-6
SM2-7A
SM2-7
SM2-8
SM2-9
SM2-10
SM2-11
SM2-11A
SM2-12
SM2-13A
SM2-13
SM2-14
ACE1
SM2-15
AvrPi7
AvrPik/kp/km
TEL 4
SM2-1
SM2-1A
SM2-2
SM2-3A
SM2-3
SM2-4
SM2-4A
SM2-5A
SM2-5
SM2-6
SM2-7A
SM2-7
SM2-8
SM2-9
SM2-10
SM2-11
SM2-11A
SM2-12
SM2-13A
SM2-13
SM2-14
SM2-15
1 Mb

## Slide 3
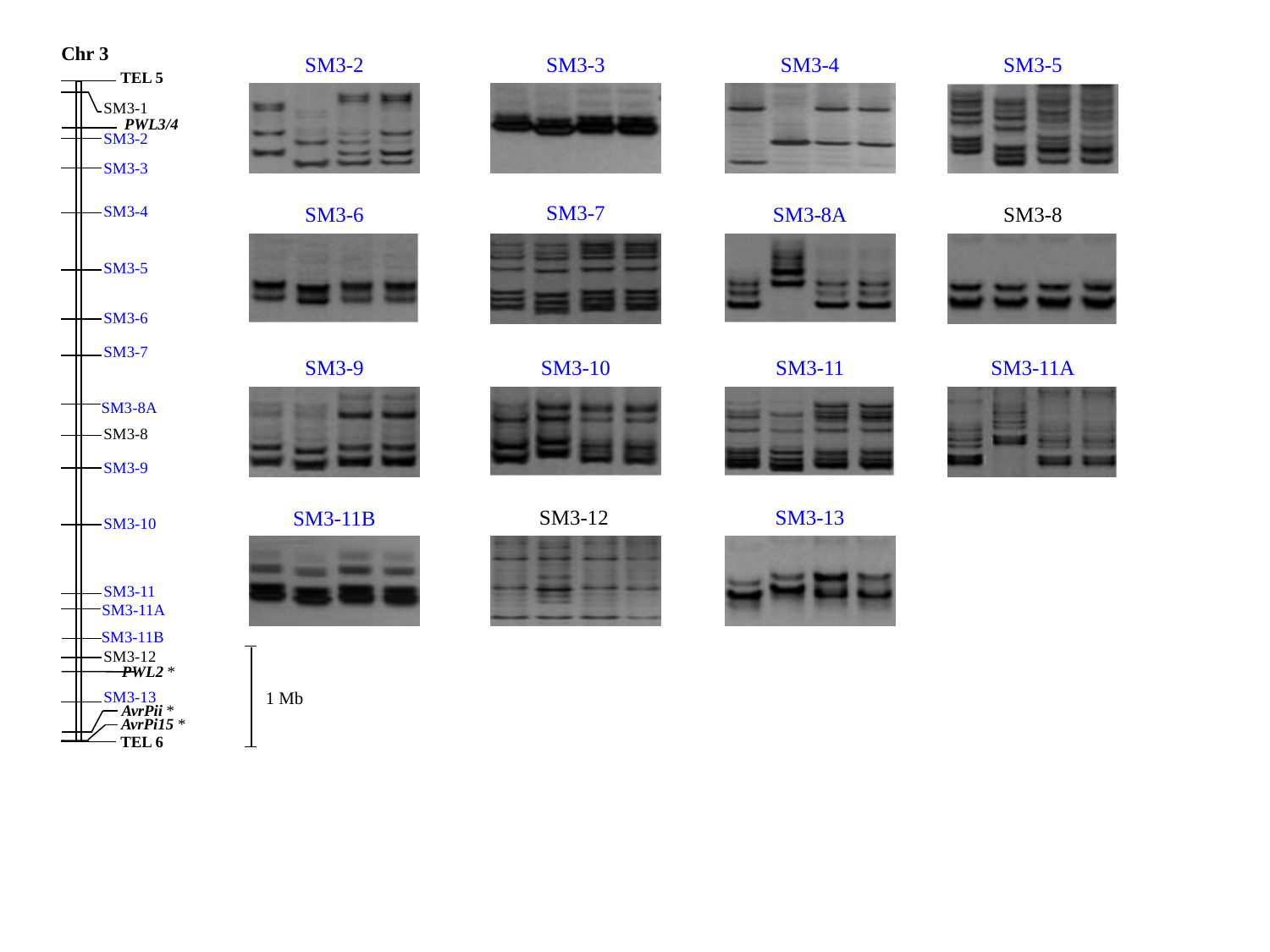

Chr 3
TEL 5
SM3-1
PWL3/4
SM3-2
SM3-3
SM3-4
SM3-5
SM3-6
SM3-7
SM3-8A
SM3-8
SM3-9
SM3-10
SM3-11
SM3-11A
SM3-11B
SM3-12
PWL2 *
SM3-13
AvrPii *
AvrPi15 *
TEL 6
SM3-2
SM3-3
SM3-4
SM3-5
SM3-6
SM3-7
SM3-8A
SM3-8
SM3-9
SM3-10
SM3-11
SM3-11A
SM3-11B
SM3-12
SM3-13
1 Mb

## Slide 4
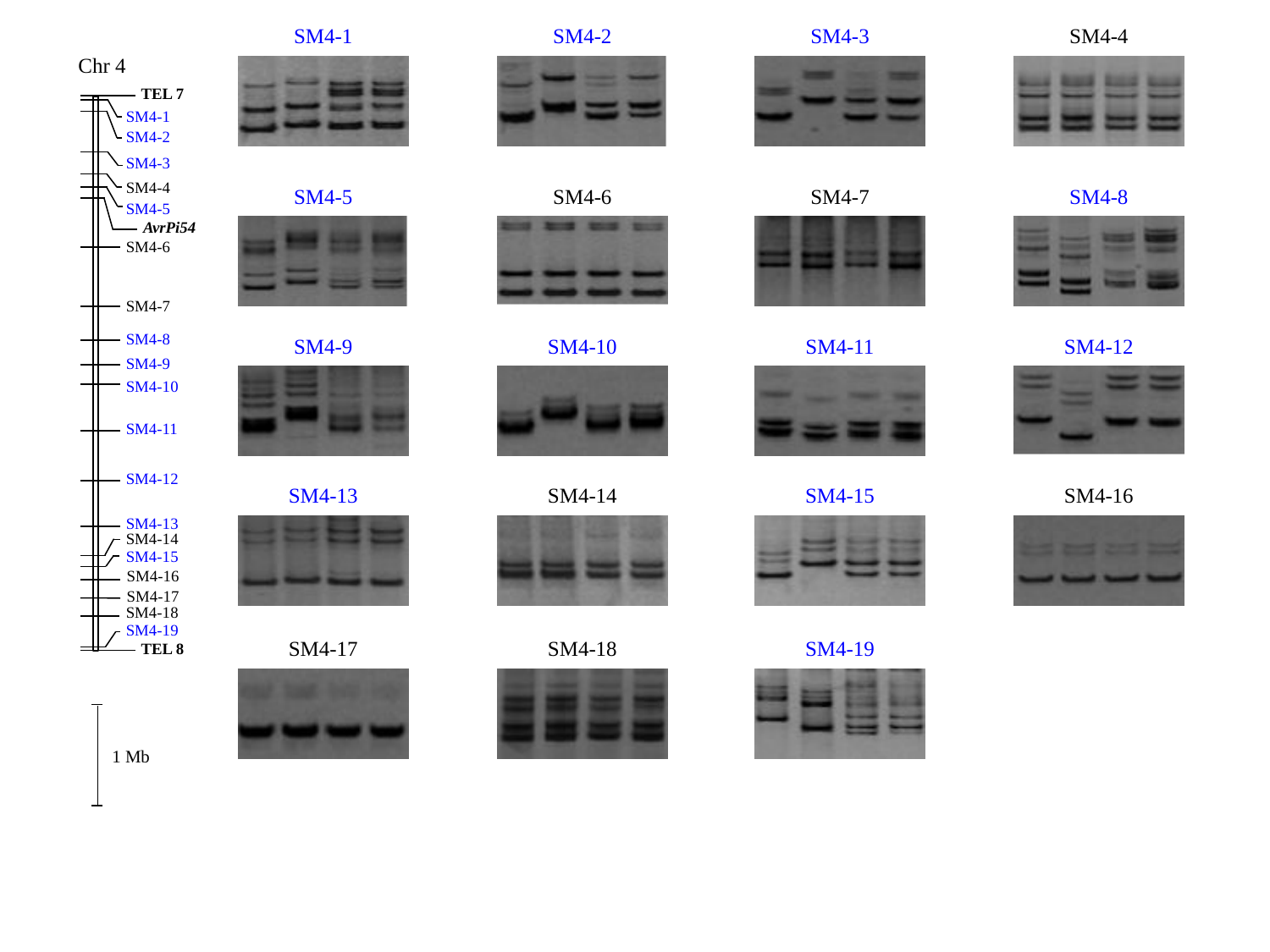

SM4-1
SM4-2
SM4-3
SM4-4
SM4-5
SM4-6
SM4-7
SM4-8
SM4-9
SM4-10
SM4-11
SM4-12
SM4-13
SM4-14
SM4-15
SM4-16
SM4-17
SM4-18
SM4-19
Chr 4
TEL 7
SM4-1
SM4-2
SM4-3
SM4-4
SM4-5
AvrPi54
SM4-6
SM4-7
SM4-8
SM4-9
SM4-10
SM4-11
SM4-12
SM4-13
SM4-14
SM4-15
SM4-16
SM4-17
SM4-18
SM4-19
TEL 8
1 Mb

## Slide 5
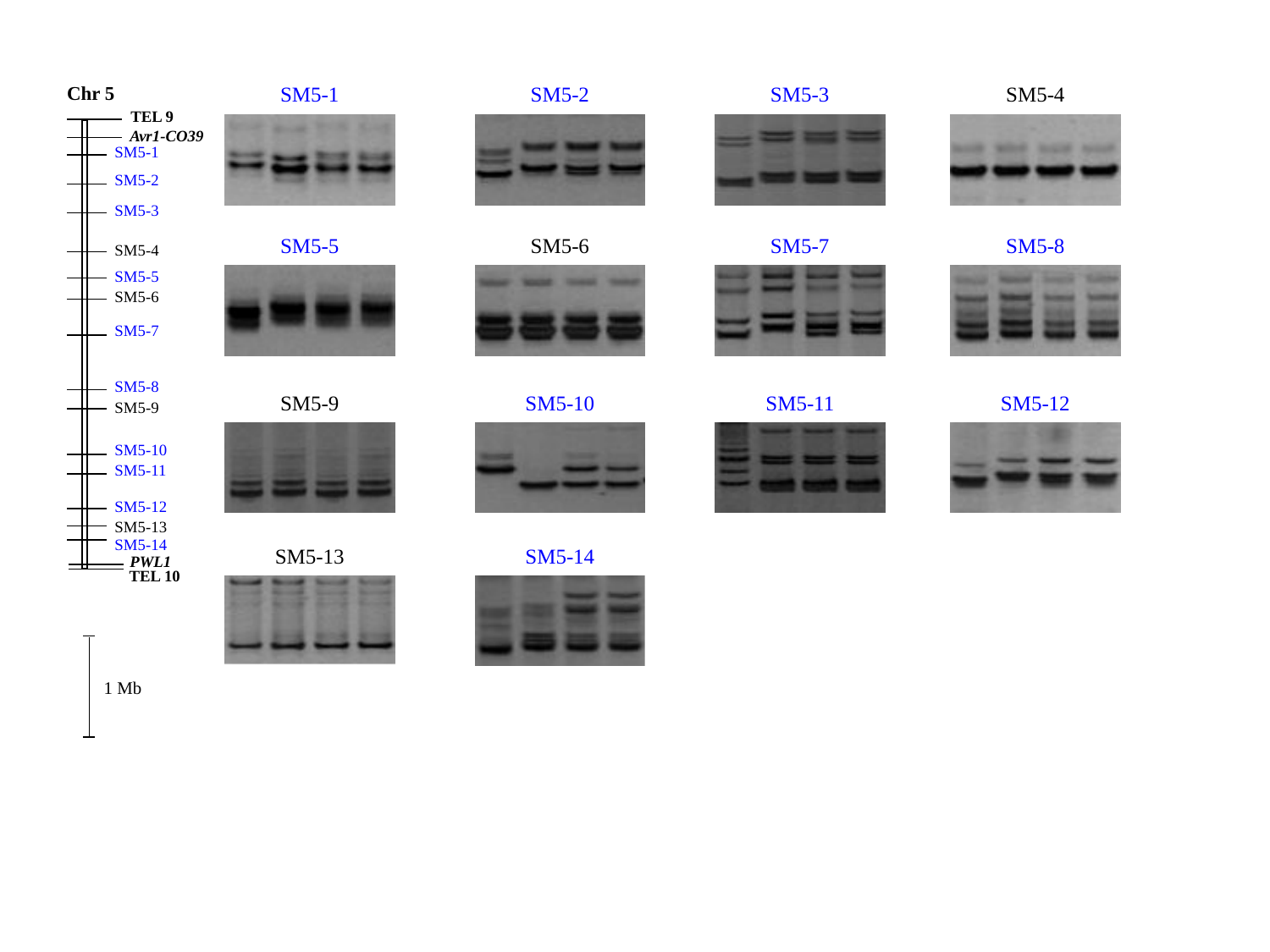

SM5-1
SM5-2
SM5-3
SM5-4
SM5-5
SM5-6
SM5-7
SM5-8
SM5-9
SM5-10
SM5-11
SM5-12
SM5-13
SM5-14
Chr 5
TEL 9
Avr1-CO39
SM5-1
SM5-2
SM5-3
SM5-4
SM5-5
SM5-6
SM5-7
SM5-8
SM5-9
SM5-10
SM5-11
SM5-12
SM5-13
SM5-14
PWL1
TEL 10
1 Mb

## Slide 6
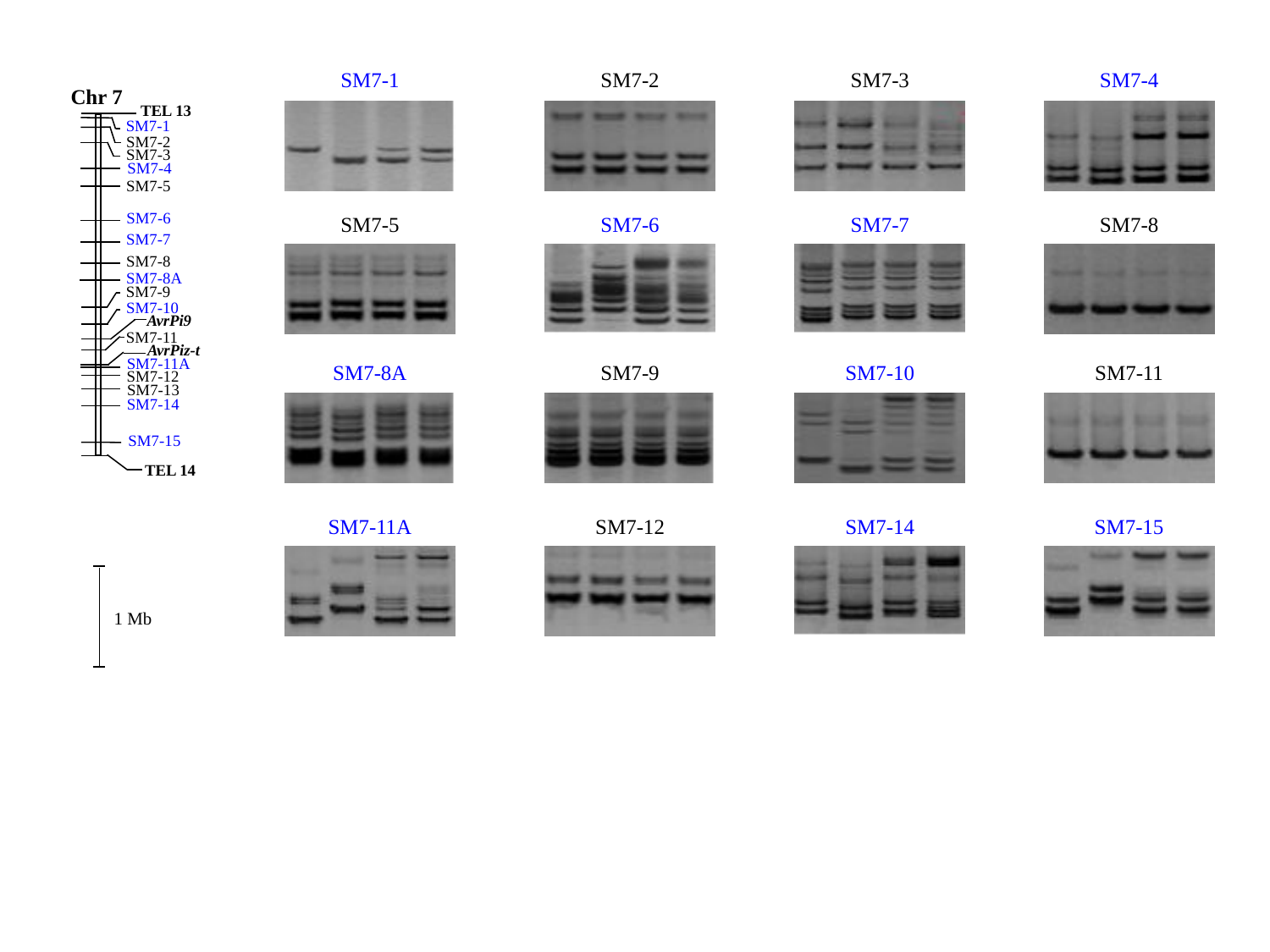

SM7-1
SM7-2
SM7-3
SM7-4
SM7-5
SM7-6
SM7-7
SM7-8
SM7-8A
SM7-9
SM7-10
SM7-11
SM7-11A
SM7-12
SM7-14
SM7-15
Chr 7
TEL 13
SM7-1
SM7-2
SM7-3
SM7-4
SM7-5
SM7-6
SM7-7
SM7-8
SM7-8A
SM7-9
SM7-10
AvrPi9
SM7-11
AvrPiz-t
SM7-11A
SM7-12
SM7-13
SM7-14
SM7-15
TEL 14
1 Mb

## Slide 7
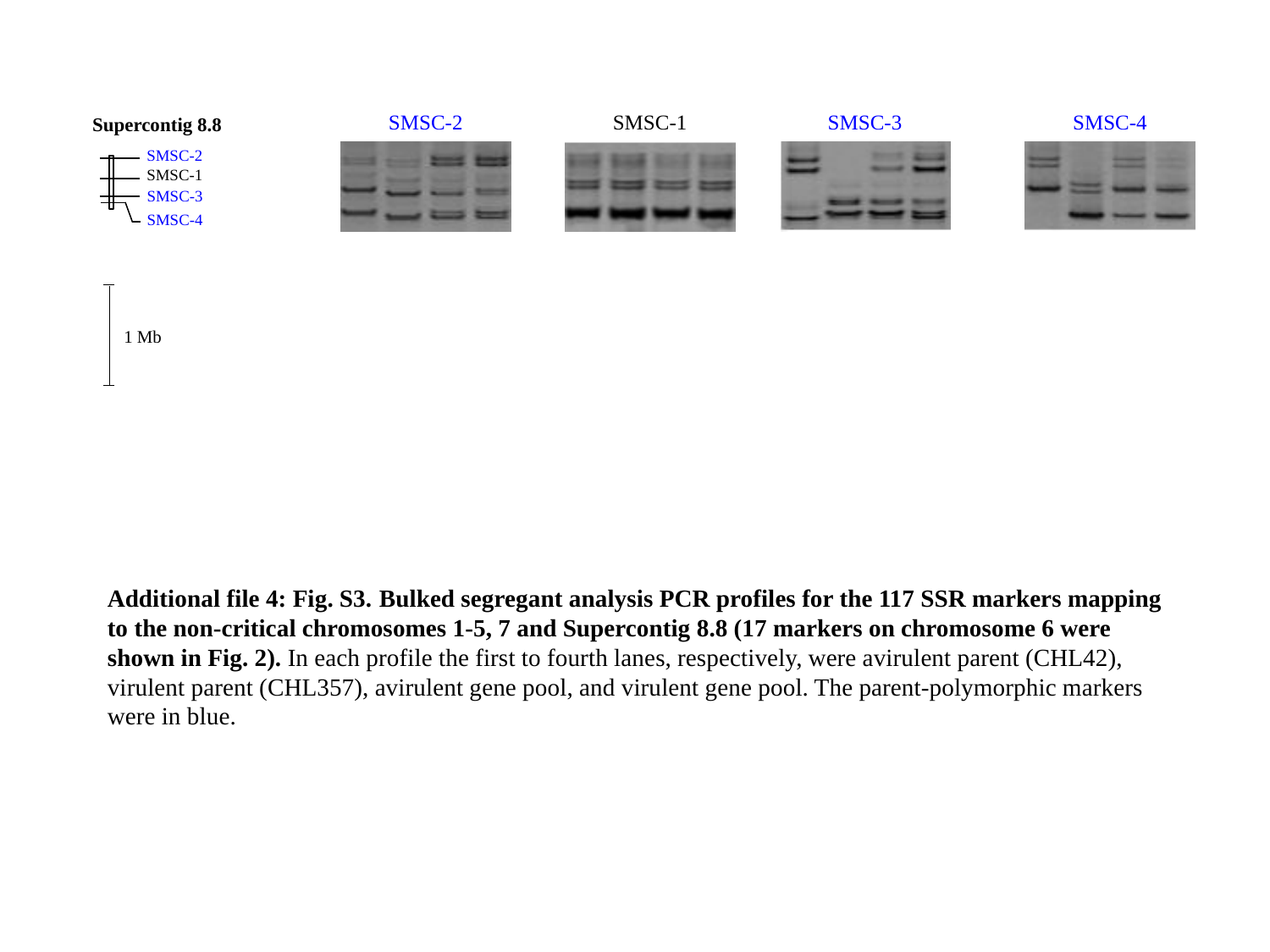

SMSC-2
SMSC-1
SMSC-3
SMSC-4
Supercontig 8.8
SMSC-2
SMSC-1
SMSC-3
SMSC-4
1 Mb
Additional file 4: Fig. S3. Bulked segregant analysis PCR profiles for the 117 SSR markers mapping to the non-critical chromosomes 1-5, 7 and Supercontig 8.8 (17 markers on chromosome 6 were shown in Fig. 2). In each profile the first to fourth lanes, respectively, were avirulent parent (CHL42), virulent parent (CHL357), avirulent gene pool, and virulent gene pool. The parent-polymorphic markers were in blue.
