# Supplementary material for: Reconstruction of an SSR-based Magnaporthe oryzae physical map to locate avirulence gene AvrPi12
Source: BMC Microbiol. 2018 May 31;18:47. doi: 10.1186/s12866-018-1192-x (PMC5984427; doi:10.1186/s12866-018-1192-x)
Supplement: Supplementary file 5 — Table S2 Candidate genes for AvrPi12 that were predicted in the target region flanked by ZSM6 and TEL12 (DOCX 22 kb) [file 12866_2018_1192_MOESM5_ESM.docx]

**Additional file 5: Table S2.** Candidate genes for *AvrPi12* that were predicted in the target region flanked by ZSM6 and TEL12

| Gene  code | Annotated  gene ^a^ | Predicted protein function domains | Secreted | Predicted  effector probability | Amino acid |
| --- | --- | --- | --- | --- | --- |
| 1 | [MGG_09795](http://fungi.ensembl.org/Magnaporthe_oryzae/Gene/Summary?db=core;g=MGG_09795;tl=udBSVnrgIWri3HcX-18025119-308630736) | PLAC8 domain | No | 0.994 | 211 |
| 2 | [MGG_09794](http://fungi.ensembl.org/Magnaporthe_oryzae/Gene/Summary?db=core;g=MGG_09794;tl=27742x5yeySpfSSh-18025122-308630830) | NA | No | 0.74 | 301 |
| 3 | [MGG_14154](http://fungi.ensembl.org/Magnaporthe_oryzae/Gene/Summary?db=core;g=MGG_14154;tl=3MF9ux9eoMwZcctV-18025146-308631516) | [Transmembrane region](javascript:domWin(1)) | No | 0.941 | 108 |
| 4 | [MGG_15429](http://fungi.ensembl.org/Magnaporthe_oryzae/Gene/Summary?db=core;g=MGG_15429;tl=9flviQV7TKYKlMfi-18025154-308634017) | Coiled coil region + [DUF4140](http://pfam.xfam.org/family?id=DUF4140) + [DUF4139](http://pfam.xfam.org/family?id=DUF4139) domain | No | 0 | 614 |
| 5 | [MGG_09793](http://fungi.ensembl.org/Magnaporthe_oryzae/Gene/Summary?db=core;g=MGG_09793;tl=8iHNGCGhogfEq2fX-18025192-308636666) | [DJ-1_PfpI](http://pfam.xfam.org/family?id=DJ-1_PfpI) domain | No | 0.015 | 249 |
| 6 | [MGG_15430](http://fungi.ensembl.org/Magnaporthe_oryzae/Gene/Summary?db=core;g=MGG_15430;tl=fzbXRIgH8aQ3wlVd-18025156-308634053) | [Signal peptide](javascript:domWin(1)) (1-20) + Glycosyl hydrolase family 10 + Fungal-type cellulose-binding domain | Yes | 0 | 559 |
| 7 | [MGG_09791](http://fungi.ensembl.org/Magnaporthe_oryzae/Gene/Summary?db=core;g=MGG_09791;tl=EFSNIYrz2PSYVkcr-18025162-308635308) | Transmembrane region | No | 0.004 | 254 |
| 8 | NA | [HTH_psq](http://pfam.xfam.org/family?id=HTH_psq) + Putative DNA-binding domain in centromere protein B + DDE superfamily + Coiled coil region + ZnF_C2HC domain | No | 0 | 556 |
| 9 | NA | [RVT_1](http://pfam.xfam.org/family?id=RVT_1) + zf_C2H2 domains + rve + Chromatin organization modifier domain + Coiled coil region | No | 0 | 1695 |
| 10 | NA | NA | No | 0.275 | 38 |
| 11 | [MGG_17845](http://fungi.ensembl.org/Magnaporthe_oryzae/Gene/Summary?db=core;g=MGG_17845;tl=HQ0GrcD8S1OmgEDG-18025203-308639037) | NA | No | 0 | 279 |
| 12 | NA | DEAD-like helicases superfamily | No | 0 | 935 |

^a^ Candidate genes were predicted based on the reference genomic sequence of isolate 70-15 via the gene predictors, FGENESH (<http://linux1.softberry.com>), GENSCAN (<http://genes.mit.edu/GENSCAN.html>), SignalP 3.0 (<http://www.cbs.dtu.dk/services/SignalP-3.0/>), SMART (<http://smart.embl-heidelberg.de/>) and EffectorP (<http://effectorp.csiro.au/>). NA, not available.
